# Multistate Markov model for household’s food insecurity transitions and their influencing predictors during COVID-19 pandemic in Ethiopia

**DOI:** 10.1371/journal.pone.0326854

**Published:** 2025-07-03

**Authors:** Henok Wariso Waqo, Gezahegn Mekonnen Woldemedihn, Zeytu Gashaw Asfaw

**Affiliations:** 1 Department of Statistics Hawassa University, Hawassa, Ethiopia; 2 Department of Epidemiology and Biostatistics, School of Public Health, Addis Ababa University, Addis Ababa, Ethiopia; Federal University of Agriculture Abeokuta, NIGERIA

## Abstract

Food insecurity is one of the most widespread social problems that millions of the world’s people are experiencing worries. The problem has been rising, particularly in Ethiopia, since 2020 due to Covid-19 with changing households’ food insecurity dynamics which is not clear yet. This study aimed to investigate the dynamics of households’ food insecurity transitions and assess the associated covariates during Covid-19 pandemic in Ethiopia*.* The study used Ethiopia-High Frequency Phone Survey longitudinal data set collected by World Bank with the total of 13517 observations repeated over time. Multistate Markov model was employed to model the paths of changing household’s food insecurity process and identify the influencing covariates of each transition. The result indicated that out of households observed in food secure state initially at one visit, 26.0% were observed in food insecure states. The corresponding values for households that were initially at mild, moderate, and severe states were 68.6%, 88.3%, and 94.8% respectively. Once a household enters mild and moderate, his incidence rate increases revealing that a household initially at severe food insecure states experiences higher risk of staying as food insecure. Sex of household head is significantly associated with food insecurity; with female headed households are respectively 1.2345 and 1.1720 times more likely to enter from food secure to mild and from mild to moderate states as compared to male headed households. Households’ residence, income loss, employment status, and assistance provisions during Covid-19 were significant predictors of food insecurity given that they were primarily at food secure and mild food insecure states; but they lack the significance for households initially at moderate state. The initial food insecurity state of households, time duration, and varying effects of covariates are the crucial issues in predicting food insecurity dynamics. Timely interventions are necessary for the effective recovery of household’s food insecurity.

## 1. Introduction

Food insecurity remains a significant global issue, with 29.6% of the global population being food insecure in 2022, 391 million more than in 2019. Global hunger is far above pre-pandemic levels, with 9.2% facing chronic hunger in 2022 compared to 7.9% in 2019 [1]. Food insecurity and nutrition reflected the period 2019−2022 as a particular moment in history with Covid-19 pandemic, economic rebound, Ukraine war, and soaring food prices impact on food insecurity differently across countries. In particular, the policy measures taken to ease the outbreak of Covid-19 pandemic such as lockdowns, social distancing, and restrictions on food supply chain have caused shocks in income and employment exacerbating the existing food insecurity [2,3].

Moreover, although the Sustainable Development Agenda aims to achieve Zero Hunger Goal by 2030, Africa has been facing a greater food insecurity problem, particularly in Sub-Saharan Africa, with hunger increasing sharply in 2020 and gentler rise in 2021; while under nutrition increased from 19.4% in 2021 to 19.7% in 2022. Ethiopia also faces a severe food insecurity crisis due to escalating violence, prolonged drought, and macroeconomic instability. The country ranks 101^st^ among 125 countries with a 26.2 score on the Global Hunger Index. Despite the government efforts, the COVID-19 pandemic, locust invasions, drought, floods, and increased food prices have made 13.6 million people food insecure [1,4,5].

In addition to Covid-19 pandemic, the emerging political unrests, unpredictable weather variability, conflicts in different part of the country, and mounting unemployment rate exacerbated food insecurity problem making many households transitioned from food secure to food insecure statuses. On the other hand, the remedial actions (such as food and non-food assistances, aid from abroad, and Safety Net Programs supporting households) taken to ease those shock event problems pulled some households to recover from being food insecure. These forces made the food security system of the country disrupted and more unstable.

Consequently, households transitioned from food secure to food insecure statuses during the pandemic and sometimes vice versa; but the exact process of transitioning, recovery, time of staying in previous food insecurity statuses, and associated covariates remains unclear.

Several previous studies carried out on food insecurity in different part of Ethiopia such as [6–11] focussed on prevalence, spatial variations, and the determinant predictors of food insecurity using cross-sectional data without considering the longitudinal effects. As to my knowledge, there are no studies undertaken on dynamics, the changing process, of food insecurity problem that Ethiopia has been experiencing during the outbreak of Covid-19 pandemic. Factors such as initial state of food insecurity a household resides, time durations, and unknown reasons can influence the rate of transition.

The limited studies that have been conducted on food insecurity transition in other countries, [12] in Nigeria and [13] in United States of America, have identified socio-demographic and food access-related factors. According [12], in Nigeria, 44.4% of households transitioned from food secure to food insecure; while 32.5% transitioned from mild food insecure to food secure status; but the dynamics of food insecurity transition is ignored. The extreme gradient-boosting machine learning model that combines machine learning, IPC ratings, and open data for more accurate forecasting was introduced [14]; but lacked acknowledging multistate transitions over time. Thus, the current study applied multistate modelling which is an increasingly popular approach outperforming other models in predictive accuracy in cases of investigating the randomly changing process among multiple state spaces.

Previous studies on households’ food insecurity transitions were also limited in their investigation of transition rates, time spent in each state, and the number of visits. In addition, they did not acknowledge that the likelihood of household entering severe food insecurity depends on his initial food insecurity status; and the possibility that the same predictors may have different effects depending up on state to state transitions and time variation. The current study employed Multistate Markov model to address these assumptions. The author believes that the dynamics of household food insecurity transitions and the influencing predictors of transitional intensities need to be better understood. So, the current study is significant as evidence-based measures from academics are crucial to combat the negative effects of food insecurity at the household level.

Besides, the two previous studies conducted [15,16] on food insecurity using the present dataset did not assess the dynamics of household food insecurity transition and state-to-state transition varying effects of predictors. The current study employed a Multistate Markov model to optimally predict household’s food insecurity statuses in Ethiopia considering factors such as initial state household resides, time duration, and non-uniform effects of covariates.

Thus, the aim of this study was to estimate transitional intensity and probability matrix, identify covariates influencing household’s food insecurity transitions, and contribute to existing literature by applying advanced statistical model for improved food insecurity prediction and targeted policy measures.

## 2. Research methodology

### 2.1 Data source and description

To assess the dynamics of food insecurity situation over time, the present study used Ethiopia-High Frequency Phone Survey of Households (EHFPS-HH) 2020−2023 dataset which was collected by World Bank (WB) as the main investigator collaborating with Ethiopian Central Statistics Agency (CSA). Besides, United States Agency for International Development (USAID), World Bank Group (WBG), and Global Financing Facility (GFF) were involved in funding process. This data was gathered as a part of WB leveraging the Living Standards Measurement Study – Integrated Survey on Agriculture (LSMS-ISA) program in 5 African countries – Nigeria, Ethiopia, Uganda, Tanzania, and Malawi. The dataset was objectively collected to support government and development partners through providing realistic data that can be used to assess the socio-economic shocks and Covid-19 pandemic implications on households. It is a panel survey data collected monthly in 15 consecutive rounds from May, 2020 to October, 2023 where completed Food Insecurity Experience of Households, the outcome variable of the current study, questions were included in five rounds (round 2, round 3, round 5, round 6, and round 11).

### 2.2 Population, sampling designs, and techniques

In this study, based on the experiences of those who are impacted, Ethiopian households comprise the study population because they accurately reflect the burden of socioeconomic issue related problems like food insecurity. Household heads were used as representative of the study subjects to get the required data from selected groups of the target population. EHFPS-HH 2020−2023 dataset was collected based on the sampling design from LSMS in 2018/2019 which was designed by Ethiopian Socio-economic Survey (ESS) that is built on nationally, regionally, residence, and gender representative sample of households in the country. As the potential impacts of the Covid-19 pandemic are expected to be severe on households’ welfare, WB team selected a subsample of households that had been interviewed for the LSMS in 2018/2019 by ESS to form multi-wave panel datasets of the country.

Finally, a sample of 3,300 households was drawn from the sampling frame of 5,374 households accessible through phones from the total of 6700 households interviewed in 2018/2019 by ESS in such a way that the sampled households are representative of the country and at regional levels; while those 1,326 households that have no phone number of their own or a reference household (friends or neighbours) were excluded from the sampling frame. Following up 3300 households, 3,107 households completed interviews in round 2; 3058 households completed interviews in round 3; 2770 households completed interviews in round 5; 2704 households completed interviews in round 6; and 1982 households completed interview in round 11. This yielded the total observation with completed interviews over time to be 13,621 from which 13,517 were measured repeatedly (at least in two rounds) and used directly for analysis of the current study.

Before conducting the final data analysis, the selection and non-response biases that could occur when sampling weights were not included was checked. There was no substantial bias driven that could potentially affect the study result. So, the effect of sampling weight was not incorporated in the analysis of this study as the selection and non-response biases based on the raw data are not likely to underestimate or overestimate real changes in food insecurity amid Covid-19 pandemic. This could be due to sampling design of ESS, used by this study, is built on nationally, regionally, residence, gender, and other groups’ representative sample of households that might avoid the disproportional selection of different population categories.

### 2.3 Study variables

#### 2.3.1 Food security and its measurements.

Food security is a dynamic concept which can be defined in the contexts of availability, accessibility to obtain appropriate foods, nutritious, and stability over time. On the other hand, food insecurity problem can occur at global, national, community, and household or individual level. This reflects its complexities in research and public policy issues which made it difficult to assess its all dimensions under a specific research work. This study assessed food insecurity at household level during Covid-19 in Ethiopia. The study variable of the current study is derived from food insecurity experience of a household where the burden of social and socio-economic implications of the pandemic and shocks lies. Thus, the current study viewed food security as the ability of households to physically and economically obtain safe and nutritious food referring to accessibility domain of food security. Although several efforts have been made so far to have single and comprehensive measure of food security, there are no single measure emerged that incorporates all dimension of food security [17,18].

Due to this, the measurements of food security used in different literatures vary and none of these measures is uniquely comprehensive enough in acknowledging the concept of multifaceted dimensions of food security. This made food security measurement to depend on the compromise of researchers based on contexts of the study rather than being absolutely perfect. Considering the context of the study, the current study used Food Insecurity Experience Scale (FIES) which measures food insecurity in various domains of food insecurity at the household level. In this context, food insecurity is defined as the condition of household not being able to freely access the food one needs to conduct a healthy, active and dignified life. From different food insecurity measurements, the current study used FIES instrument due to:

- It captures both physical and psychosocial dimensions of food insecurity; which made it the best tool for analysing food insecurity at household level.
- It is the only direct food insecurity measure from affected individuals based on the Voice of Hungry (VoH) project by Food and Agricultural Organization (FAO).
- The dataset used, HFPS-HH 2020−2023 gathered by World Bank, was surveyed in FIES; to enable evidence based decision on socio-economic implication of Covid-19.
- Food insecurity measurement by FIES is in line with measuring food insecurity by FAO, Global Report on Food Crisis (GRFC), and Sustainable Development Goal [19–21].

The list of eight standard FIES questions, each with yes or no possible response, includes:

FI1: During the last 30 days, was there a time when you or others in your household were worried about not having enough food to eat because of lack of money or other resources?
FI2: During the last 30 days, was there a time when you or others in your household were unable to eat healthy and nutritious/preferred foods because of a lack of money or other resources?
FI3: During the last 30 days, was there a time when you or others in your household ate only a few kinds of foods because of a lack of money or other resources?
FI4: During the last 30 days, was there a time when you or others in your household had to skip a meal because there was not enough money or other resources to get food?
FI5: During the last 30 days, was there a time when you or others in your household ate less than you thought you should because of a lack of money or other resources?
FI6: During the last 30 days, was there a time when you or others in your household went without eating for a whole day because of a lack of money or other resources?
FI7: During the last 30 days, was there a time when your household ran out of food because of a lack of money or other resources?
FI8: During the last 30 days, was there a time when you or others in your household were hungry but did not eat because there was not enough money or other resources for food?
FI8: Household members have not eaten all day because of lack of money or other resources?

The response of each household to each of eight FIES food insecurity questions is measured as:

$\begin{array}{c} \text{Yi}=\left\{ \begin{array}{c} \begin{array}{cc} 1\;\;\;\;\;\;\;\;\;\;\text{if\textbackslash{} a\textbackslash{} \textbackslash{} \textbackslash{} household\textbackslash{} \textbackslash{} \textbackslash{} expriences} & \;\;i^{the}\;\text{insecurity\textbackslash{} \textbackslash{} \textbackslash{} question}\\ 0\;\;\;\;\text{if}\;\text{a}\;\text{household}\;\text{doesnot}\;\text{exprience} & i^{the}\;\;\text{food\textbackslash{} insecurity\textbackslash{} question} \end{array}\; \end{array}\right.\;\; \end{array}$ for i=1,2,...,8

#### 2.3.2 Food insecurity states of households.

Food insecurity severity of households, dependent variable of the current study, was derived using discrete assignment of FIES raw scores. In this method, food insecurity severity classification is derived equivalently from the primary measure of experienced food insecurity globally [19]. Food insecurity severity classification, equivalent to global averages and cut-off points noted [19], was developed by using discrete assignment of raw scores of FIES by [20–21]. In this view, the household’s food insecurity severity statuses, here after called states, obtained by adding raw scores of eight FIES questions with cut-off points is categorized as: food secure if his FIES raw score is 0; mild food insecure if his FIES raw score is 1–3; moderate food insecure if his FIES raw score is 4–7; and severe food insecure if his FIES raw score is 8. Due to its advantage in terms of transparency and ease of explanation to the public and policy officials, discrete assignment of raw scores is the preferred method for food insecurity severity classification (state) and became a norm for within-country study [22].

Predictors of household food insecurity severity were derived based the literatures and availability of data in HFPS-HH 2020−2023 dataset. However, the number of covariates included in the current study is limited due to the number of reasons. Firstly, data on some predictors are not available in HFPS-HH 2020−2023 dataset. Secondly, since the analysis of current study is based on longitudinal dataset collected over time, it was not possible to link the dataset with previous 2018/2019 ESS survey to get records on some characteristics of households such as education level and family size unlike the studies by [15,16]. The demographic characteristics which were available in HFPS-HH 2020−2023 dataset and assessed were: sex of household head (1 = Male; 2 = Female); age of household head (1=Below 40; 2 = 40 and above); residence of household (1 = Rural; 2 = Urban); current employment status of households (0 = Not employed; 1 = Employed); taking assistance since Covid-19 outbreak (0 = Did not get assistance; 1 = Obtained the assistance); and total household income change since last call (1 = Not reduced, 2 = Reduced).

### 2.4 Multi-state model

Multi-state model is a statistical frame work used to investigate the paths of the process of the outcome variable over time allowing subjects to move among a finite number of states. It is a process (Y(u),uεt) with a finite state spaces S={1,2,....,N} where uε(0.t),t<∞ is the time interval of observation and N is the number of transitional states. According to [23], Y(t) is the stochastic process defined as:


$$Y(t)\sim MSM(\lambda_{ij}(t);\;i,j=1,2,3....N)$$ (1)

where i and j are respectively the initial and final states of household’s food insecurity in a given measurement time t. Then, the stochastic process Y(t) is governed by an intensity matrix Λ(t) with entry $\lambda_{ij}$ or by a transition probability matrix P(t) with entry$p_{ij}$ whose raw sum to 1. The current study viewed household food security transition as randomly changing stochastic process where next food security status of a household depends on his current status. To acknowledge such process in the analysis of household food security transitions, this study used Multistate Markov model based on [24,25].

**Fig 1:**
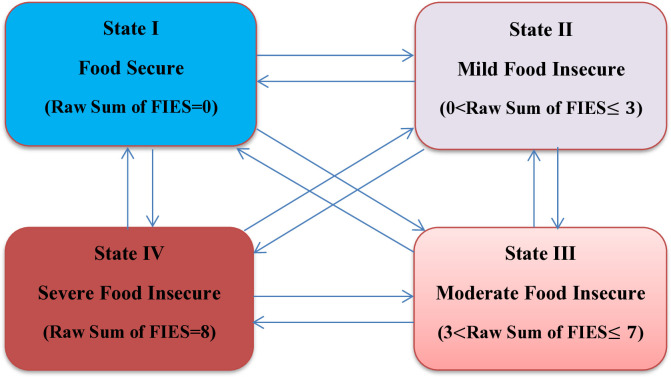
Multistate transition model of a household food insecurity severity over time.

Households experience transition among four food insecurity state spaces; namely, food secure state (state 1); mild food insecure state (state 2); moderate food insecure state (state 3); and severe food insecure state (state 4) ordered representing severity of their food insecurity experience. All the states are transient due to the fact that whether or not a household or its member dies, the household continues in experiencing food insecurity problem over time. Besides, direct transitions food insecurity jumping the state/s between exiting and entering states is allowed assuming the existence of different factors for the event of interest. Accordingly, in the current study, household’s food insecurity transition is considered as multi-state processes illustrated using diagrams with boxes representing four states spaces and arrows between the states representing the possible transitions as depicted in Fig 1.

#### 2.4.1 Parameters of transitions in multistate model.

**Baseline transitional intensity:** The rate of transition to more severe food insecure state j for the household currently in less severe state i based on [25,26] is given by:


$$\lambda_{ij}(t,F_{t})=\underset{\delta t\to 0}{\lim}P(\frac{X(t+\delta t)=j/X(t)=i}{\delta t})$$ (2)

$\lambda_{ij}$: represents the instantaneous risk of moving from state i to state j and form a matrix Λ

X(t): is the outcome variable (state) occupied by a household at time t.

X(t+δt): is the outcome variable (state) occupied by a household at time t+δt (small

change in time)

Assuming a time-homogeneous Markov process, the transition intensity matrix is


$$\Lambda =\left[\begin{array}{cccc} {}*20c-(\lambda_{12}+\lambda_{13}+\lambda_{14}) & \lambda_{12} & \lambda_{13} & \lambda_{14}\\ \lambda_{21} & -(\lambda_{21}+\lambda_{23}+\lambda_{24}) & \lambda_{23} & \lambda_{24}\\ \lambda_{31} & \lambda_{32} & -(\lambda_{31}+\lambda_{32}+\lambda_{34}) & \lambda_{34}\\ \lambda_{41} & \lambda_{42} & \lambda_{43} & -(\lambda_{41}+\lambda_{42}+\lambda_{43}) \end{array}\right]$$

**Transitional probability matrix:** For the current study, the transition probability matrix representing a household in food insecurity state i at initial time t_0_ will be at state j at time t_1_ (after time t duration) with entries $p_{ij}$ is given by:


$$P(t)=\left[\begin{array}{cccc} {}*20cp_{11} & p_{12} & p_{13} & p_{14}\\ p_{21} & p_{22} & p_{23} & p_{24}\\ p_{31} & p_{32} & p_{33} & p_{34}\\ p_{41} & p_{42} & p_{43} & p_{44} \end{array}\right]$$

where i and j are the current and next food insecurity states respectively (i = j = 1,2,3, 4) and $p_{ii}$: is diagonal element of transition probability matrix showing staying in the initial state.

Besides, this study analysed total time (the average time a household is expected to stay in non-absorbing state i at the end of the follow up period t), sojourn time (the expected length of time that a household stay in a state i before he transits to next state j, and the expected number of each state i is re-visited (recurrence).

#### 2.4.2 Multi-state regression models.

One drawback of baseline transition is that it does not tell us the contribution of the covariates as it corresponds to hazard setting all values of covariates to zero. In a multi-state model, assessing the effect of constant or time-varying characteristics of individuals to their transition rates is often of interest which is mostly investigated by modelling the intensity as a function of the predictors. To identify the influence of household characteristics on the transition rate of food insecurity, the current study applied proportional intensity model where the transition intensities Λ(t,Z);1≤i<j≤4) is the function of duration of time and covariates as:


$$\lambda_{ij}(t,z)={\lambda_{ij}}^{\left(0\right)}(t)\exp(\beta_{ij}^{T}Z)$$ (3)

where ${\lambda_{ij}}^{\left(0\right)}(t)$ is the baseline intensity function between states i and *j*, $\beta_{ij}^{T}$ is the vector of regression parameters, and *Z* is the covariate vector. For Homogeneous and piecewise Non Homogeneous Model, this study used Cox proportional hazard model based on [27]; which assumes changes in predictors produce proportional changes in the hazard. Thus, ${\beta_{ij}}^{\prime }s$ relating the transition intensities with covariatesZ are constant over time as given by:


$$\lambda_{ij}(z)=\lambda_{ij}\exp(\beta_{ij}^{T}Z)$$ (4)

The effect of the covariates were evaluated based on multistate Cox “proportional hazards” regression whose standard form assumes the hazards for any two households have the same proportion at all times. In this case, the portion of transitional intensity for each transition from state i to state j determined by covariates is the log linear function of the covariates. Predicting future outcomes from a MSM with time-dependent covariates acknowledges changes in the covariate due to change in time. Assuming time-dependent regression coefficients$\beta_{ij}(t),$ an alternative model, proposed by [28] for survival data and used by current study to construct transition probability matrix considering time-dependence effect of covariates is given by:


$$\lambda_{ij}(t,z)={\lambda_{ij}}^{\left(0\right)}(t)\;+\beta_{ij}^{T}(t)Z$$ (5)

### 2.5 Covariate selection for transitions in multistate regression model

In multistate modeling, there are three common methods of choosing covariates in targeting which transitions are affected by which covariates. These are covariates on rates of specific transitions, covariates on all transition rates, and identical effects of the same covariates on different transitions. The current study employed modelling covariates on specific transitions targeted to state transitions for which notable occasions of data information are available in the frequency distribution. Then, the study fitted the null model with no covariates and four optional models with all covariates included; and selected the model with smallest AIC for extracting the final result of the analysis.

### 2.6 Predicting transitional intensity in multistate regression model

Inclusion of covariates to the multistate model may increase or decrease the transitional rate entries of Λ(t) matrix depending up on their relative effect on the specific transition. Those covariates having direct association increase the magnitude of the corresponding entry while those that are associated indirectly decrease the incidence rate. The current study made prediction of household food insecurity risk at three (mean; increasing food insecurity risk, and decreasing food insecurity risk) values of the covariates.

### 2.7 Ethical approval and consent to participate

This analysis uses publicly available and deidentified data collected by the World Bank (HFPS-HH 2020–2023, available via the World Bank Microdata catalogue: https://microdata.worldbank.org/index.php/catalog/3716/get-microdata). As such, this analysis is exempt from ethical approval. However, the original data collection obtained ethical clearance and all participants completed appropriate informed consent procedures, prior to participating in the survey. In addition, only consented surveys were kept in the dataset with all personal identifying information dropped from the clean dataset. Additionally, authors obtained a letter of consent for secondary analysis from College of Natural and Computational Science Research Ethics Review committee of Hawassa University, on May 22, 2023 with RERC reference number: CNCS-REC 025/23.

## 3. Results

### 3.1 Descriptive results

In this study, 3300 selected representative sample of households interviewed in five follow up periods in which Food Insecurity Experience Scale questions were included yielded the total observation measured over time longitudinally (at least in two rounds) to be 13517. The analysis was conducted accordingly and decisive results were presented. To explore and visualize the possible flows of households’ food insecurity over time, the current study used spaghetti plot, box plot, average observation times of households’ food insecurity, and the observed frequency of households in consecutive follow ups.

**Fig 2:**
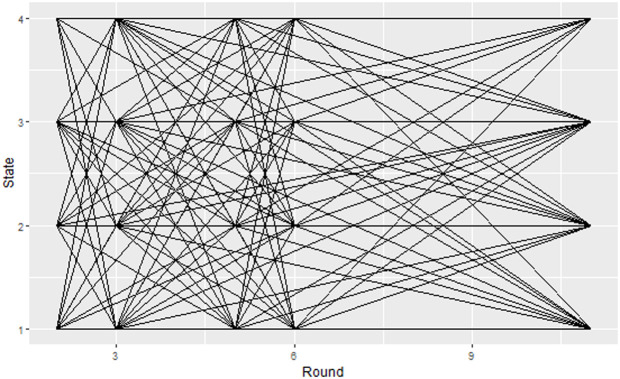
Household’s food insecurity movement viewed during five follow up measurements.

In Fig 2, the horizontal lines along a given state represent households staying in the initial state; while none horizontal lines between states represent the progression of households to other states; where the severity of household food insecurity increases from state 1 to state 4. The result from Fig 2 clearly revealed that households are likely to enter in to and exit from all states of food insecurity over time.

#### 3.1.1 Observation times of households’ food insecurity during the follow ups.

In the current data, the fixed times of follow ups corresponding to round 2, round 3, round 5, round 6, and round 11 during which food insecurity experience questions included were 0.000 (base line), 0.7667, 3.4667, 4.3667, and 11.3667 in month’s period since study entry (baseline assessment). During five rounds follow ups, the median and mean time households food insecurity measurement made were 3.4667 and 3.4073 months respectively.

#### 3.1.2 Observed frequency of households at different states of food insecurity over time.

The result from Table 1 indicated that, from 4415 households observed at food secure state (state 1) initially, 26.0% were observed at food insecure states, with 20.1% at adjacent state (state 2); 5.5% and 0.4% at state 3 and state 4 respectively. In this case, the majority (74%) of households initially at food secure state stayed at their previous state. Out of 2983 households in state 2 initially, 68.6% stayed at food insecure states (state 2, state 3 and state 4) with larger occasions at their previous (mild) state and 31.4% were observed at food secure state (recovery). The percentages of households observed at food insecure states for households that are initially at state 3 and state 4 were 88.3% and 94.8% respectively. So, Table 1 result indicates the proportion of households observed at food insecure states (state 2, state 3, and state 4) increases as the severity of the initial state of household food insecurity increases and provides data information that directs further assessment of the predicting covariates of the transitions.

**Table 1:** Frequency distribution of pairs of consecutive observed states.

From state	To state				Total households initially
	State 1	State 2	State 3	State 4	
State 1	3267(74.0%)	886 (20.1%)	245(5.5%)	17(0.4%)	4415
State 2	937(31.4%)	1419(47.6%)	598(20.0%)	29(1.0%)	2983
State 3	305(11.7%)	671(25.8%)	1449(55.7%)	176(6.8%)	2601
State 4	20(5.2%)	48(12.4)	180(46.8%)	137(35.6%)	385

Note: Table 1 summarizes the observed number of households in one food insecure state at one visit and each other state at the next visit. The frequencies do not reflect the progression of household food insecurity; but only the observed occasions.

**Fig 3:**
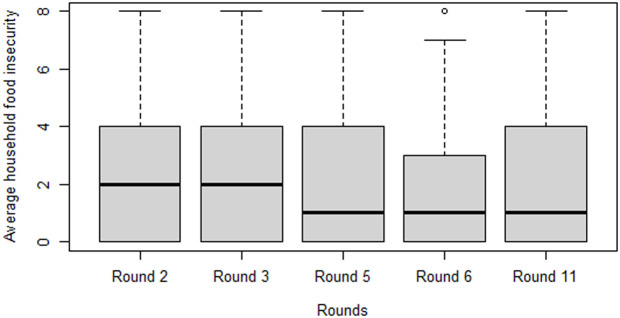
Median households’ food insecurity experience measured by FIES in five rounds.

Fig 3 shows the average (median) number of food insecurity questions in which household experienced food insecurity during each round. Box plot in Fig 3 indicated that the severity of household’s food insecurity in the first two rounds is higher than the last three rounds. In the first two rounds the median value is around 2 revealing households approximately experienced food insecurity in two of eight food insecurity questions; while the corresponding values of last three rounds are around 1 showed smaller household food insecurity experience during the letter rounds.

### 3.2 Estimates of baseline transitional intensities and the associated parameters

#### 3.2.1 Baseline transitional intensity.

The result from Table 2 pointed out that the rate of transition of households initially at food secure state (state 1) to mild insecure state (state 2) was 0.273804 with corresponding transitions to moderate and sever state 0.001641 and 0.000326 respectively. This indicates that the larger transition from state 1 is made to its adjacent (mild state) with slight possibility of direct transition to state 3 and state 4. Once households entered to state 2, they could transit to forward and backward adjacent states with the rate of 0.322576 and 0.440019 respectively with the rare (0.000148) direct transitions to state 4. After households entered in to state 3, they experienced higher (0.116817) rate of transiting to the most severe state (state 4). Households initially at state 4 either stayed in the state or transited to adjacent moderate insecure state with larger rate 0.766246; but with least probaibilty (0.002548) of the direct transition to food secure state (recovery). The result has revealed that those households that were at more severe food insecure states initially had higher rate of transition to severe food insecure state. This is an indication that the past food insecurity history of household is one of the factors that determine the transition of household in to severe food insecure state.

**Table 2:** Estimates of baseline transitional intensities.

State-State transition	Baselines intensity	Confidence interval for the baseline intensity
State 1 – State 1	−0.275772	(−0.302500, −0.251400)
State 1 – State 2	0.273804	(0.234200, 0.320100)
State 1 – State 3	0.001641	(0.000211, 0.012740)
State 1 – State 4	0.000326	(0.000212, 0.000519)
State 2 – State 1	0.440019	(0.395800, 0.489100)
State 2 – State 2	−0.762743	(−0.860400, −0.676200)
State 2 – State 3	0.322575	(0.265800, 0.391500)
State 2 – State 4	0.000148	(0.000025, 0.000864)
State 3 – State 1	0.020105	(0.008272, 0.048870)
State 3 – State 2	0.394426	(0.351400, 0.442700)
State 3 – State 3	−0.531349	(−0.576900, −0.489400)
State 3 – State 4	0.116817	(0.093990, 0.145200)
State 4 – State 1	0.002548	(0.001717, 0.003780)
State 4 – State 2	0.002232	(0.000336, 0.014820)
State 4 – State 3	0.766246	(0.639300, 0.918400)
State 4 – State 4	−0.771026	(−0.919400, −0.646600)

Note: The baseline instantaneous transition rates with their corresponding 95% confidence intervals displayed in Table 2 are the default values obtained by setting the values of covariates at zero.

#### 3.2.2 Transition probabilities.

According to the results from Table 3, household at food secure state initially had approximately 0.2008 probability of being food insecure after a month (with probability 0.1708 of being at mild; 0.0286 of being at moderate, and 0.0014 of being at sever food insecure states). Once households entered to state 2, they had 0.7226 probability of staying as food insecure domains (mild; moderate, and sever food insecure states) after a month’s period; while the remaining 0.2774 probability was being recovered to food secure state. A particular household initially at the moderate food insecure state had 0.9334 probability of being in food insecure domain: with 0.2209 at mild; 0.6495 at moderate; and 0.0630 at severe food insecure states. Given that a household entered severe food insecure state, there was 0.9796 probability of being in food insecure states within a month with very small (0.0204) probability of being in food secure state (recovered). This indicated that as household gets in to more severe food insecure states, it would be very difficult to recover to food secure state. From 4-month probability in Table 3, the probability that a household at food secure state initially would be food insecure after 4-month was 0.4252; while the equivalent value after 1- month was 0.2008. Once households entered in to mild food insecure state, they would experience 0.5579 probability of staying in the food insecure state domains after four month with 0.4421 probability of recovering to food secure state. However, household at moderate food insecure state (state 3) initially experienced larger probability (0.7004) of staying as food insecure states (state 2, state 3 and state 4) and smaller tendency (0.2996) of recovering to food secure state. Regarding households initially at severe food insecure state, they would experience 0.7839 probability of staying at food insecure states and 0.2161 probability of recovering.

**Table 3:** Estimated 1-Month and 4-month transitional probability matrices.

Raw-column state transition probability	1-month transition probability matrix				4-month transition probability matrix			
	State 1	State 2	State 3	State 4	State 1	State 2	State 3	State 4
State 1	0.7992	0.1708	0.0286	0.0014	0.5748	0.2699	0.1399	0.0154
State 2	0.2773	0.5343	0.1785	0.0099	0.4421	0.2945	0.2310	0.0324
State 3	0.0666	0.2209	0.6495	0.0630	0.2996	0.2897	0.3482	0.0625
State 4	0.0204	0.0807	0.4134	0.4855	0.2161	0.2647	0.4099	0.1093

Note: The values in the Table 3 are 1-month and 4-month probabilities for household’s backward (recovery) and forward (incidence) food insecurity transitions and staying in his previous position (diagonal values). The values are obtained considering the dynamics of initial food insecurity state the household resides and time duration until next observation made.

**Fig 4:**
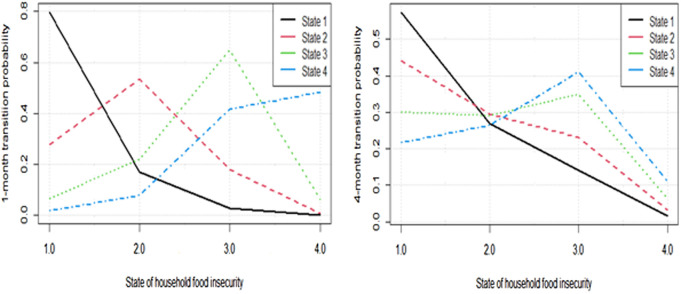
1-month and 4-month transition probability paths.

When the time of household food insecurity prediction changes from 1-month to 4-month probability, two notes are made. Firstly, similar to 1-month transition probability, the severe food insecurity of households initially the higher the risk of being more severe food insecure next follow up and vice versa. Secondly, as time goes from 1-month to 4-month, unlike 1-month transition probability, the probability that households at food secure state initially would face less probability to stay as food secure(increased risk) and those households initially at more severe food insecure states experience larger probability to transit to less severe states of food insecurity (increased recurrence). The changes in 1-monh to 4-month prediction probability paths of households’ food insecurity transition from each state can be viewed from Fig 4.

#### 3.2.3 Time of staying and visits of household food insecurity states.

Results from Table 4 indicated that households that are initially at food secure state (state 1) approximately stayed the total of 7 months in the state they were previously residing (state 1) over the 12 months study period. Once the households get in to food insecure states, they spend shorter periods: approximately 3 months in mild; 2 months in moderate and quarter of a month in severe food insecure state. This showed that the severe the initial state of households the shorter total time they stay. Regarding the sojourn time spent by household, a particular household in food secure state (state 1) stayed 3.6 months with SE 0.156 before transition to food insecure states. The expected length of time that a household stays in mild, moderate, and sever food insecure states in a single stay are respectively 1.315, 1.892 and 1.298. These result has revealed that households are more stable (stay for larger time periods) in their residing state when they are at food secure state (state 1) and move from one state into another with in shorter time periods once they enter in to mild, moderate, and severe food insecure states. Considering the expected number of months (recurrence) that a household visits mild food insecure (state 2) was higher, approximately 2 months and 18 days, than visiting other states. This might be due to the state can be considered as bridge for the households that transit between food secure state and other food insecure states (moderate and severe sates).

**Table 4:** Total time, sojourn time, and number of visits (recurrence).

States	Total time of stay	Sojourn time	Standard error	Recurrence
State 1	6.9071	3.6262	0.1710	1.3781
State 2	3.0478	1.3111	0.0806	2.6052
State 3	1.8090	1.8820	0.0790	1.1754
State 4	0.2361	1.2970	0.1165	0.2140

Note: Table 4 presents total time of stay (the estimated length of time that a household at each state spent in 12 months study period), sojourn time (the mean length of time that a household stay in a state i before he transits to next state j, and recurrence (the expected number of time that a household visits each state.

#### 3.2.4 Model assessment for base line transition rates.

**Fig 5:**
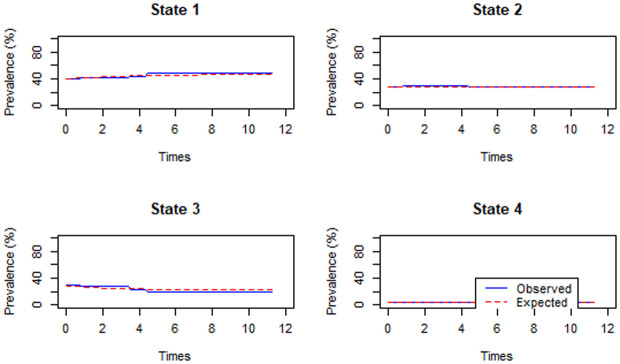
Model assessment for baseline transition rate of household food insecurity.

The result from Fig 5 depicted that there is no considerable discrepancy between observed and expected percentages in all states except in state 4 where the event of food insecurity is underestimated between 5–10 months. Based on [26], this is an indication that multistate model fitted the data for the current study.

### 3.3 Model and covariates selection in multistate modelling of food insecurity transition

The result in Table 5 indicated that the null model and four optionally fitted multistate regression full models had different values of AIC, measure of predictive ability of a model. Then, model 2 with the smallest AIC representing forward transition to adjacent states was chosen as a part of inference. This model was used to identify covariates influencing a household to enter more severe adjacent food insecurity states; namely, to transit from food secure state to mild food insecure state; mild food insecure state to moderate food insecure state; and moderate food insecure state to severe food insecure state.

**Table 5:** Model adequacy and selection.

Over all Test of the model	Log likelihood	df	AIC
Null model without any covariate	−10080.3060	12	20184.6100
Optionally fitted full (covariates included) models with different possible transitions			
Model 1 (All forward possible transition)	−9993.4650	32	20050.9300
Model 2 (Forward to adjacent state transition)	−9990.8370	30	20041.6700
Model 3 (Backward transition or recovery)	−10020.7080	30	20101.4200
Model 4 (Forward and backward transition)	−9975.4880	48	20046.9800

Note: The overall test of the model comparing AIC of null and four full models. Abbreviations: AIC, Akaike’s information criterion; df, degree of freedom.

### 3.4 Results on estimates of multistate regression coefficients

As indicated in Table 6, for household initially at food secure state (state 1), the hazard ratio corresponding sex of household head was 1.2345 (1.0848, 1.4050). The entire Confidence Interval (CI) lied above 1 indicating that female headed household is significantly associated with the increased risk (1.2345 times more likely) of transition from state 1 to state 2 as compared to male headed household holding other effects constant. Regarding households primarily at state 2, female headed households experienced the increased hazard with 1.172 times more likely to enter moderate state; whereas for households at moderate state initially, male and female headed households do experience statistically the same risk of transition to severe food insecure state (state 4). There is slight possibility that households aged above 40 were significantly associated with less risk of entering in to moderate food insecurity provided that they were initially at mild food insecure state keeping other predictors constant. Otherwise, age of household head does not have significant association with likelihood of household’s food insecurity. Residence, getting assistance, and income loss are variables that are associated significantly with the incidence of food insecurity only if households are in state 1 and state 2 initially. Those households obtaining assistances and living in urban are associated with decreased incidence of food insecurity; while those whose income reduced since Covid-19 pandemic are associated with increased risk. In the contrary, these variables had no significance influence on food insecurity once household enter moderate food insecure state. As evidenced in the Table 6, the hazard ratio corresponding to employment status for household initially at food secure state is 0.8578 (0.7429, 0.9904); which pointed out that those household employed during Covid-19 would experience less risk of entering to adjacent (mild) food insecurity state; otherwise, employment status was not significantly influencing them to enter to adjacent more severe food insecure states.

**Table 6:** Estimates of multistate regression parameters.

Covariates	State 1 – State 2	State 2 – State 3	State 3 – State 4
Sex of household head (reference = male)			
Female	1.2345 (1.0848,1.4050)	1.1720 (1.0097,1.3600)	0.9981 (0.7443,1.3380)
Age of household head (reference <40 year)			
HH head aged ≥ 40	1.0200 (0.9075,1.1463)	0.8656 (0.7544,0.9931)	0.8896 (0.6788,1.1658)
Residence of household (reference = rural)			
Urban household	0.6958 (0.6056,0.7994)	0.7223 (0.6198,0.8416)	0.8515 (0.6368,1.1386)
Employment of household (reference = not employed)			
Employed households	0.8578 (0.7429,0.9904)	0.9502 (0.8042,1.1227)	0.8505 (0.6139,1.1784)
Assistance of household (reference = no assistance)			
Assisted household	0.6236 (0.4645,0.8370)	0.7175 (0.5434,0.9473)	0.7285 (0.4555,1.1651)
Income loss of household after Covid-19 (reference = not decreased)			
Household with reduced income	1.1938 (1.0497,1.3580)	1.4959 (1.2992,1.7220)	0.9084 (0.6938,1.1890)

Note: The values in Table 6 represent multistate regression parameters estimates in terms of Hazard Ratio (HR) that indicates each corresponding variable as increasing or decreasing food insecurity incidence rate/hazards of a household. The estimate greater than 1 with entire Confidence Interval (CI) falling above 1 indicates the corresponding predictor has statistical significant influence in increasing the hazards; while those with HR less than 1 with entire CI below 1 indicates the corresponding predictor has significant influence in decreasing the hazards of household’s food insecurity. However, those estimates whose CI include 1 irrespective of their magnitudes show insignificance of the corresponding variable in influencing the hazards of households’ food insecurity.

In general, with exception of age of household head, all the included covariates were significantly associated with hazard rate of food insecurity provided that the households were at food secure (state 1); and once household entered in to moderate state, the predictors were not significantly associated with the risk of food insecurity.

### 3.5 Prediction of food insecurity incidence setting covariate values

As evidenced in Table 7, the hazards with covariates set at their means for households primarily at state 1; state2; and state 3 are 0.3022; 0.3212; and 0.1149 respectively. The respective predicted values when all covariates are set at values that decrease the risk of food insecurity are 0.2294; 0.2200; and 0.1022. These are the rates of transitions for a male headed household; aged above 40; living in urban; whose income did not reduce after Covid-19; get employed and obtaining assistance during Covid-19 for a household initially at respective states. All these intensity rates are less than their corresponding baseline hazards obtained by setting covariate values at zero (Table 2) and baseline hazards obtained by setting covariate values at their means (Table 7) because all covariates entered in to the model had decreasing effect of food insecurity risk. However, when all covariates are set at values that increase the risk (female headed; aged below 40; living in rural; income reduced after Covid-19; did not get employed; and with no assistance), the hazard rates are 0.8906; 0.9050; and 0.1975 provided that a household is initially at state 1, state 2, and state 3 respectively. Irrespective of the initial state of households, the incidence rates predicted setting covariate values at increasing hazard of food insecurity are greater than their respective baseline hazards in Table 2 and Table 7.

**Table 7:** Predicted transitional incidence rates at the given values of the included covariates.

All included covariates	State to state hazard ratios		
	State 1 – State 2	State 2 – State 3	State 3 – State 4
Set at their mean values	0.3022 (0.2740, 0.3349)	0.3212 (0.2884, 0.3577)	0.1149 (0.0938, 0.1398)
Set at decreasing household’s food insecurity hazard	0.2294 (0.1992, 0.2642)	0.2200 (0.1830, 0.2646)	0.1022 (0.0739, 0.1431)
Set at increasing household’s food insecurity hazard o	0.8906 (0.6229, 0.1274)	0.9050 (0.6438, 0.1272)	0.1975 (0.1069, 0.3648)

Note: Predicted state to state food insecurity incidence rates (forward transitions) with corresponding confidence interval in brackets for covariates set at their mean, decreasing, and increasing values of household’s food insecurity.

### 3.6 Results with time-dependence consideration of covariates

Although the model assessment indicated multistate model fitted the data well, there is slight underestimation in state 4 from 5–10 months period as displayed in Fig 5 that might be due to time varying covariates. To assess the magnitude of the effect, the estimated 4-month transitional probability matrix obtained by acknowledging time dependence of covariates is shown in Table 8. The diagonal entries of Table 8 is slightly smaller than the corresponding entries of 4-month predicted transitional probability matrix obtained without considering time dependence of predictors in Table 3. This shows that the larger probability of transitions among different states occurs when time variation of covariates is considered. However, the corresponding entries of household food insecurity transition probabilities are not considerably different.

**Table 8:** 4-month transitional probability matrix with time dependence of covariates.

Raw-column state	State 1	State 2	State 3	State 4
State 1	0.5379	0.2780	0.1618	0.0203
State 2	0.4515	0.2907	0.2237	0.0341
State 3	0.3452	0.2912	0.3062	0.0574
State 4	0.2731	0.2779	0.3589	0.0901

Note: The values in the Table 8 are 4-month probabilities for household’s backward (recovery) and

forward (incidence) food insecurity transitions considering the time dependence of the covariates.

## 4. Discussion

This study assessed transition of household food insecurity and its associated predictors in Ethiopia during a special moment in history of socio-economic problems and food shortage due to Covid-19 pandemic and other shock event manifestations. Several studies conducted on food insecurity in the country supported the finding of the current study. However, the findings of this study are unique as it estimated household’s food insecurity transition rate and its associated covariates acknowledging the past history of household’s food insecurity and the state to state transition varying effects of the predictors which was hardly investigated in the country. Thus, the findings of the current study lay the foundation for direct future interventions to account for non-static process of households’ food insecurity and acknowledge their food insecurity history and time durations so as to make optimal food insecurity prediction.

According to the results of the study, the direct forward (hazard) and backward (recovery) transition jumping the state between are possible. This contradicts [29] that for ordered state spaces, representing severity of event of interest, transitions are allowed only between adjacent states. This might be due to the phenomena of Covid-19 and shock events may make households to transit directly from state 1 to state 3 or state 4 instantaneously. On the other hand, the interventions may enforce them to instantaneously recover directly from severe food insecure to food secure state.

Households primarily at the more severe food insecurity states experienced the larger rate of progression to severe state indicating that the current household’s food insecurity status matters for his next being of food insecure which exactly applies to Markov assumption. On the other hand, households at severe food insecure state experienced considerable recurrence. This might be due to the existence of assistance programs designed to alleviate the problem of such events during Covid-19 manifestation. The probability that a household enters food insecure states increased as time duration increased; whereas, the probability that it recovers also increased as increase in time duration. This means that increasing time duration increases the risk for households that are initially at food secure state; but increases the chance of recovery for those at food insecure states initially.

The analysis of the current study regarding covariates effects on transition provided new insights in that it examined the effect of covariates acknowledging the same covariate may have different effect on transition of household food insecurity depending up on their initial state. The finding from previous studies such as [12,16,30,31] revealed female-headed households experienced food insecurity significantly higher than male-headed ones. The current study finding is consistent with this fact in that, provided that households are at food secure and mild food insecure states, female-headed households experience higher risk of food insecurity than male-headed households during Covid-19. However, according to the current finding, there were exceptional cases that once households entered to moderate state; there was no statistically significant difference in the risk of food insecurity between female-headed and male-headed households. In contrast to study [21]; but similar to the study [15], this study has pointed out that households living in urban are less likely to experience more severe food insecurity than rural dwellers. However, according to the current study finding, once households get into moderate state, residence is not significantly associated with the risk of food insecurity. Households that were employed during study period were associated with a lower likelihood of experiencing food insecurity provided that they were at food secure state initially; which confirms with most previous studies [13,16]. However, given that households are at mild and moderate states, the finding of this study is inconsistent with those findings. Given that households were at food secure and mild food insecure states, the provision of assistance to households during Covid-19 significantly decreased the risk of food insecurity. This result is supported by the finding [32]; but the slim difference of this study is that assistance provision had no considerable effect for households at state 3 initially. The studies [11,16,33] showed that the reduction in income of households during Covid-19 have exacerbated the likelihood of their food insecurity. The current study finding confirms this fact provided that households are initially at state 1 and state 2; otherwise, income loss is not significantly associated with transition of households. For most included covariates, with exception of age of household head, the way the risk of more severe food insecurity is associated with households at food secure and mild food insecure states initially was the same; while for households that are at moderate state initially, most of the included covariates were not significantly associated. This may be due to, when households enter to moderate state; they are highly viable to severe food insecurity and covariates might lack considerable influence.

The current study finding indicated, 4-month food insecurity probability prediction obtained by acknowledging time dependence of covariates yield slightly larger transition rates as compared to the case of time dependence of covariates is not considered. This small change might be due to considering time effect on time-dependent covariates reduces the probability that a household stays in his initial position (stability of the system) and hence increases his transition. However, the increment in transition is not considerable due to time period of the study is short (12 months) during which most covariates might not be notably affected.

In general, this study considered food insecurity history of household, the pace of the transition, time durations, and the varying effects of the covariates which were not covered by the previous studies using multistate Markov model. If these issues are ignored, the inference will be less valid leading to formulation of less effective policy measures of food insecurity problem of the country. However, due to absence of data, the study was limited to include some important predictors of household characteristics and commonly occurring shock events (displacements, drought, and flood) during Covid-19 that might adversely affected food security situation of the country. In order to completely comprehend the relationship between food insecurity and other disruptive events in the nation, comprehensive data on the phenomena of shock events and the measures implemented to lessen such events are required.

## 5. Policy implications

The finding of this study is important because it incorporates the complexities of food insecurity problem that has not been addressed by the previous studies using advanced statistical model and found out the important results that can be used for the effective policy measures and targeted intervention strategy. In particular, this study indicated that households are at different (mild food insecure, moderate food insecure, and severe food secure) states of food insecurity with initial household’s food insecurity status significantly matters in determining its future food insecurity status. The policy implication of this result is that any intervention made to mitigate food insecurity problem needs to consider not only the number of food insecure households; but also their initial food insecurity statuses. So, while implementing Productive Safety Net, Household Building programs, and others humanitarian aids to build households capacity during shocks events, considering the severity statuses (mild food insecure, moderate food insecure, and severe food insecure) of household’s food insecurity is the necessary intervention option.

The result also revealed that the same predictors have different influencing power for households at different food insecurity states. In addition, predictors of food insecurity lacked significance for households after it entered moderate food insecurity. Thus, intervention strategies should consider the varying effect of the predictors for state to state transitions. Especially, a timely intervention before food insecure households enter in to severe states of food insecurity is the best policy option for the effectiveness of the intervention. If policy measures are not designed in this ways, working on those predictors will not impact statistically significant improvements on the status of household food insecurity. Besides, the adverse effects of shock events such as drought due to unpredictable weather condition and displacements due to political unrests in the country are emerging problems making the economic system more unstable which increases the dynamics of food insecurity transitions over time. So, during planning and execution, government and development planners need to give due considerations to the possible risks of unexpected shock event manifestations that might aggravate the disruption of households’ food insecurity. Besides, promoting cultural socio-economic life of the people where they support each other during shock events can be a first aid to prevent households from entering severe problems. In summary, the current study may lend support to realistic intervention and policy formulation, which demand top priority.

## 6. Conclusions

In conclusion, initial food insecurity statuses of households and time duration are the crucial factors of their future food insecurity risk. Household’s food insecurity predictors have varying effects depending up on the state to state transitions. Once households enter a moderate state, covariates lose power and the interplay between predicting covariates and food insecurity likelihood diminishes. Policy measures designed to curb food insecurity problem should take in to account the history of households’ food insecurity and time duration of its prediction. To bring significant improvement of household’s food insecurity, timely interventions before they enter severe food insecure state is necessary. The influence of household’s food insecurity predictors should not be considered as uniform effect; rather, it has to be considered as varying effect depending up on the past food insecurity history of households.

## Abbreviations

AIC: Akaike’s Information Criteria
ESS: Ethiopian Socio-economic Survey
FAO: Food and Agricultural Organization
FIES: Food Insecurity Experience Scale
HFPS: High Frequency Phone Survey
LSMS: Living Standards Measurement Study
MSM: Multi-state model
UNICEF: United Nations Children’s Fund
WB: World Bank
WHO: World Health Organization.
